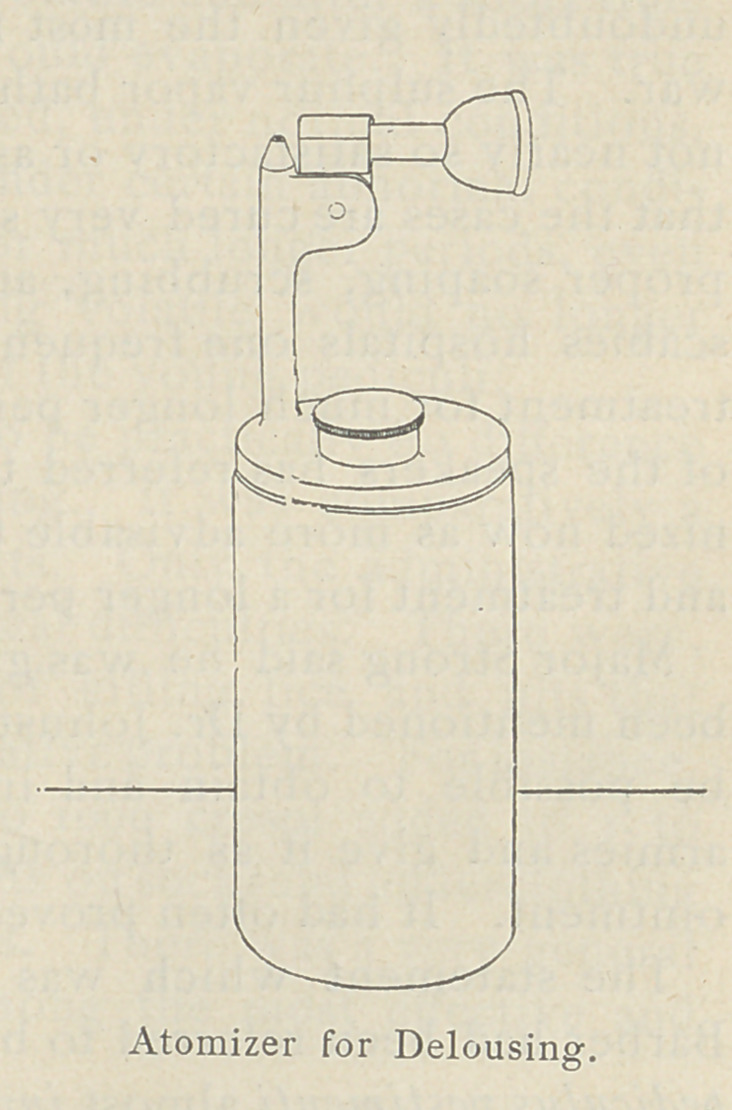# The Second Session of the Research Society of the American Red Cross in France

**Published:** 1918-02

**Authors:** 


### The Medical Bulletin

N° 4 PARIS FEBRUARY 1918

### RESEARCH SOCIETY REPORTS

### THE SECOND SESSION OF THE RESEARCH SOCIETY OF THE AMERICAN RED CROSS IN FRANCE

January 14 and 15, 1918, at 6, rue Piccini, Paris.

MAJOR LAMBERT, Chief Surgeon of the American Red Cross in France, presided throughout the session. The subject of the first meeting was Tetanus.

COLONEL SIR WILLIAM LEISHMAN, R. A. M. C., opened the subject by giving a short account of the incidence of the disease in the British Armies on the Western Front, from the commencement of the operations to the present time.

He pointed out that the introduction of compulsory prophylactic inoculation of antitoxin after all wounds, however slight, had enormously reduced the frequency of tetanus and he said that he would limit his remarks on this very large subject chiefly to the use of tetanus antitoxin, first from the preventive side and then from the therapeutic.

1. *The Prophylactic Use of Tetanus Antitoxin.* — As soon as it was realised how heavily the soil in the battle zone was impregnated with Tetanus spores, orders were issued for the universal application of prophylactic inoculations, at the earliest possible moment after the receipt of the wound. At first it was considered — as far as information on the subject was available — that 500 U. S. A. units was a sufficient dose, and the speaker still thought it was sufficient in the majority of wounds. But in instances of severe injury, where the wounds were large or deep and were heavily contaminated, and especially when they were accompanied by fracture of bone, he thought the dose of 500 units was not so good as one of 1000 or 1500 units. Accordingly the first orders on the subject were modified and, in all cases of the character described, medical officers were directed to give the larger dose. There were also those unfortunate cases of men who had lain out in No Man's Land for two or three days. In all such cases it had been directed that 1500 units were to be given without delay and that if there was any special reason to anticipate tetanus, antitoxin treatment should be commenced forthwith.

Another application of the prophylactic employment of antitoxin was in cases of trench foot. As soon as it was realised that cases of tetanus were liable to occur among men with trench foot, — and very grave cases they often were, — orders were issued that any man suffering from this condition who showed any blistering or other lesion of the skin should receive a prophylactic dose of antitoxin. This had a very pronounced effect, but in spite of it cases of tetanus continued to occur in which there could be found no obvious evidence of a lesion. This dose was therefore made compulsory in every case of trench foot, irrespective of the presence of a lesion, and every man received an inoculation of tetanus antitoxin. Since that order came into effect it had practically stamped out this grave complication.

With regard to the repetition of the prophylactic dose, the speaker stated that Sir David Bruce and the Tetanus Committee in England, over which he presided, had recommended that the first prophylactic dose should be repeated three times, at intervals of from 7 to 8 days. In France their recommendation had been adopted, with a reservation, however, in the case of men with very slight wounds who needed to be kept only a few days in hospital and who were then passed on to a convalescent camp or back to duty. If such cases were retained in hospital until they had received all four doses of prophylactic antitoxin, it would lead to considerable waste of military service, In such slightly wounded cases only the second prophylactic dose was given and there was no evidence of any harm having resulted from this procedure. All the graver cases, however, now received their four doses of prophylactic serum; the first as soon as possible after being wounded, the second, third, and fourth at intervals of seven days. As the majority of our wounded were sent to England when fit to travel the third and fourth doses were most frequently given there.

Statistical proof of the increased amount of protection given by the multiple doses, as compared with the single dose was as yet lacking; the speaker's own figures were inconclusive and, as he understood from Sir David Bruce, he too was not yet in a position to answer this question. On theoretical grounds, however, it was obviously sound policy to maintain the antitoxin concentration in the blood over the period during which danger might be anticipated.

*The prophylactic use of antitoxin.* — Referring to late or secondary operative interference with a wound, the speaker said there was a good deal of evidence in British experience, and in the published experiences of other armies, that there existed considerable danger of such secondary operations' stirring up a latent focus of infection, especially in wounds involving fracture of bone, or those in which pieces of clothing or other foreign bodies might have escaped the notice of the surgeon. Tetanus spores might be lying dormant in such a wound and the second operation might give them a chance of growth and the man might develop tetanus. That pointed clearly to the advisability of giving a prophylactic dose of antitoxin prior to any such delayed operation and this, as the speaker understood, was coming into more frequent use and was certainly sound policy.

2. On the subject of the therapeutic application of tetanus antitoxin, he said we were on much more uncertain ground. One would think that at this stage of the war we should be able to state definitely what degree of good the specific treatment of tetanus had done, what was the best channel by which to give it, and what was the best system of dosage. He did not think we were in that position yet. He had collected British records in France and had studied them very closely, with the assistance of Major Smallman; and Sir David Bruce and his Committee had done the same in England, and they all had sought for answers to these questions. There appeared to be no clear answers yet. The problem was a very difficult one. From innumerable laboratory experiments a great deal was known about the effects of antitoxin on experimentally infected animals, the rate of absorption, and the fate of antitoxin inoculated by various paths; but it was not known how far in the wounded man the tetanus toxin had already poisoned the nerve centers, nor to what extent the out-pouring of fresh tetanus toxin was still going on. The dose of our remedy was known, but not the dose of poison we were asking that remedy to neutralize.

Any attempts to contrast the alternative methods of dosage were greatly complicated by possible fallacies of many kinds. Naturally the wounds differed immensely in gravity and the man's condition was influenced by many factors, such as the existence of gas-gangrene, severe sepsis, shock, or loss of blood, quite apart from the tetanus and the treatment employed for it. Death might also occur from another complication when the tetanic symptoms were being mastered or even, apparently, cured. These were but a few of the factors which made comparison of particular groups of cases treated on different systems an extremely difficult problem.

All possible effort had been made to contrast such groups and eliminate the more obvious fallacies. The impressions gained from this procedure in France as to the relative value of the various routes of inoculation, — they were more impressions than conclusions, — had led the observers to attach more value to the intramuscular and subcutaneous channels than to the intrathecal and intravenous. With these views Sir David Bruce and his Committee were not in accord as they considered that repeated intrathecal injections should hold the first place among the four alternative channels by which one might introduce the antitoxin : the subcutaneous, the intramuscular, the intrathecal, and the intravenous. The intraneural route and the local application to the wound of antitoxin, either fluid or desiccated, had been little employed. Figures and analyses appeared to indicate that the channels by which the antitoxin was more slowly absorbed, the intramuscular and the subcutaneous, had given better results on the whole than those by which the antitoxin was more quickly absorbed, the intravenous and intrathecal. On the other hand there was a large amount of experimental data, and also of clinical experience in certain cases, which would appear to point to the value of the intrathecal method, especially if employed early. The main idea of the intrathecal injection was that by introducing the serum directly into the spinal canal you may get it into close contact with the poisoned cells of the central nervous system, and that by saturation of these cells with antitoxin you might be able to dissociate from them some of the toxin they had already absorbed. Experiments had not as yet demonstrated very clearly the possibility of this being accomplished, but some recent experimental work done in England by Professor Sherrington had shown, in the case of monkeys, that an intrathecal injection could save the animal from a fatal dose of tetanus toxin, whereas intramuscular and subcutaneous injections mostly failed to do so.

Advocates of the intravenous method were not so common and this method was the one in which the dangers of anaphylactic shock were greatest. This was unfortunate as it was obviously the most rapid and thorough way by which to saturate the whole of the blood and tissues with antitoxin. No case in France treated by this channnel only had recovered.

The other methods were slower. By the subcutaneous route the antitoxin was not all absorbed from the site of inoculation for about 48 hours, while the intramuscular route, though quicker, demanded 24 hours. They had the advantage however of appearing free from risk, so that very large doses might be given and a continuous action of the antitoxin might be maintained. It appeared that in the larger proportion of recoveries one or both of these methods had entered largely into the treatment employed. The figures, however, were open to many interpretations and the observers were still searching for fresh light on this subject.

The question of the dosage of antitoxin was an equally difficult one. There was, however, general agreement on three points: that a high dosage was necessary, that it should be commenced as early as possible, and that its use should be maintained well into convalescence. And yet cases were known where the dosage had been colossal without saving the patient, and severe cases in which in spite of doses which appeared altogether inadequate the man recovered.

The speaker said he feared the impression he must have given was that we had still a very great deal to learn of the treatment of tetanus by antitoxin, but there was, at least, no doubt left in our minds as to the great benefit of the prophylactic dose, it had almost stamped out the disease from among our wounded men. There was, however, great difficulty in proving the therapeutic value of tetanus antitoxin.

Major Lambert asked the speaker whether any relation between the incubation period and the severity of the disease had been observed. He also asked if serum had been injected in the sciatic nerve. He requested Sir Bertrand Dawson to state whether symptoms in war conditions differed from those observed in civil practice.

Sir Bertrand Dawson, R. A. M. C., replied that he did not think they did. Some curious manifestations, however, had been observed in the early stages of tetanus. Purely local rigidity in a limb might be remarked in the case of a man who was not very ill. In the general run of cases, however, there was nothing new.

Major Lambert remarked that local manifestations were conspicuous in laboratory animals but that almost invariably the local symptoms became generalized. He had noticed in human beings that the recti muscles were the first to become stiffened and the last to relax. A great many cases that were sent into medical wards as rheumatism proved to be tetanus. He called upon Major Murphy, to open the discussion.

Major Murphy, U. S. R., stated that he had seen very little tetanus thanks to the care with which the British tetanus prophylaxis was administered. He believed that most of the cases that he had observed had been due to some slip in applying the prophylaxis. Either the second dose had not been given exactly on time or an emergency operation had been attempted without giving the usual preventive dose. He believed that local tetanus sometimes occurred, but very often what appeared to be local tetanus might be nothing more than a spasm of muscle due to the local irritation. His experience in treatment had been just as discouraging as Sir William Leishman would indicate. Cases seemed to get well in spite of treatment rather than because of it. He believed that in the present zeal for large doses of antitoxin, we were inclined to forget what our fathers knew of the advantages of sedatives in carrying tetanus patients through. We had not, perhaps, used the sedative sufficiently.

MAJOR ROBERTSON, U.S.R., said that he believed there was hardly any other disease or any other bacillus about which so much was known, and yet about which, beyond the limits of that knowledge, so many questions could be raised.

The speaker stated that he had made a careful examination of the soil from all parts of the Western Front, and that wherever it had been cultivated it was infected to such an extent that if only a grain of it were inoculated into a laboratory animal, it would invariably cause tetanus. We should remember that every soldier had his body as well as his clothing saturated with tetanus infected soil. Even a clean wound might be seriously infected. Thus many of the mysterious cases might be explained.

He believed that the delay that frequently intervened between the time of injury and the injection of the prophylactic dose, had been responsible for a large incidence of tetanus.

Since by the subcutaneous route it took from 24 to 36 hours for the serum to be absorbed in the blood stream, where alone it could become effective, it was highly important that means should be devised for giving the serum immediately upon the receipt of the wound.

The speaker stated that the suggestion had been made that cotton or gauze, upon which antitoxin had been poured and dried, might serve as a first aid dressing that could be immediately applied, and so prevent the onset of the tetanus until the proper prophylactic injection could be given. In laboratory experiments, such a pad was found to be effective against three or four times a fatal dose of toxin. In applying these pads to actual cases they could be moistened in water or saline solution. The idea seemed to the speaker worthy of a trial.

On the question of the second dosage of antitoxin the speaker stated that experiments upon laboratory animals had shown a lessening of the period of immunity given by each successive inoculation. After the first prophylactic injection, the immunity conferred lasted from 12 to 15 days at its maximum, and in a decreasing degree for 21 days. The second dose, however, was quickly eliminated by the body, as any foreign substance might be, within from 7 to 8 days at its maximum. The third dose was effective for an even shorter period.

Theoretically, then, by repeating doses we were reducing the patients' ability to hold the antitoxin in the blood. Thus the prophylactic dose administered before an operation might not afford protection during a long incubation period following the operative wound.

In laboratory experiments it had also been observed that when the toxin had been absorbed in the blood stream it was, in the case of guinea-pigs, conveyed to the *lower* part of the spinal cord. In the case of human beings, horses, rabbits, etc., it often went to the upper part of the spinal cord. This appeared to be a purely selective action. Another very interesting fact observed was that histological examination of the spinal cord during and after tetanus failed to show the slightest evidence in cells or tissues as to where the tetanus toxin was actually working. When these animals recovered, the recovery was complete. It would appear, therefore, that the toxin, although it is bound to injure the cells, does so only temporarily, and that, if life could be prolonged for a sufficient period, the body might gradually eliminate the toxin and a normal state be restored.

Another point observed in the laboratory was the impossibility of separating toxin from the cells, if it were once bound to them. Animals had been dried out by the administration of strong saline solutions in an attempt to drag the toxin from the cells. It was impossible, however, to affect toxin that had once united with the spinal cord. Only the toxin in the blood, therefore, could be effectively combated. It did not matter, perhaps, whether the doses given were large, or whether they were frequently repeated, if only they were sufficient to neutralize the toxin in the blood and the toxin coming from the wound. If this were true, the intravenous route for therapeutic purposes was strongly indicated. The quicker the antitoxin could reach the blood stream the better, for in the meantime enough toxin might become attached to the nerve cells from the blood to produce serious results.

The speaker then urged the more general adoption of magnesium sulphate in cases of tetanus, to rest the patient from spasms which wore him out. Used subcutaneously it was an ideal preparation. A 25 o/o solution was strong enough to bring an animal near to death, but its effect could immediately be neutralized by injections of calcium chloride. The speaker believed that it was a drug which deserved abler and more vigorous use than was given it at present.

Major Blake, U.S.R., said he believed that he was one of the first to treat tetanus by magnesium sulphate. Meltzer in 1904, he believed, had discovered the effectiveness of magnesium sulphate in producing anesthesia and paralysis. About a month before this discovery was made public, Meltzer had made a personal communication of the fact to the speaker, who used it at the Roosevelt hospital to produce anesthesia in four or five cases before he gave it up. It produced anesthesia that lasted several hours, and paralysis that lasted about 24 hours. At just that time a case of tetanus with violent convulsions came to the hospital. The speaker conceived the idea that magnesium sulphate might relieve the patient and afford him a much needed rest. He had a good heart and showed little depression. The magnesium sulphate quieted the convulsions for about 24 hours. They recurred, however, and the dose (4 cc. 25 o/o solution) was repeated. The convulsions ceased for another period of 24 hours. After a third dose they recurred with less frequency and the patient got well. The patient had also received full doses of tetanus antitoxin.

Encouraged by the effect of this treatment the speaker tried it on another patient who died. Such was the result also in seven or eight other cases in which it was tried. Most of the cases were bad ones with a great deal of depression as well as exhaustion produced by the convulsions. The speaker believed that the magnesium sulphate merely added to the depression, thus hastening the end. He therefore abandoned its use.

One interesting point brought out in the discussion was that the intramuscular method of injection of antitoxin had seemed to give better results than the others. Possibly on account of its being absorbed more slowly it was not eliminated so rapidly from the body and therefore more time was allowed for the direct combination of the antitoxin with the nerve cells. If this were the case, it seemed that a method of administering antitoxin which prolonged its absorption was preferable. For example if serum were injected by the intravenous method it was probably eliminated very rapidly, while with the slower method of absorption the elimination would not be so rapid.

Since Sir Bertrand Dawson and Major Murphy had raised the question of local tetanus, the speaker wanted to give his observations on this point. What were apparently local spasmodic contractions were observed in the case of a man with a comparatively slight fracture of the elbow. He was convinced at first that these were merely muscular spasms. Weights were applied, but the painful, recurrent contractions persisted. On the administration of the antitoxin he got well almost immediately. The speaker had observed another very similar case, in which recovery followed the use of antitoxin. In these cases it appeared that the antitoxin had a real effect. The speaker believed that practically all such cases recovered. He said that Captain Taylor had made some interesting observations in this connection with regard to the persistence of bacteria in old wounds.

Captain Taylor, U. S. R., said he supposed the observations referred to by Major Blake were those made during experiments carried out on bone sequestra from cases some of which were completely healed, and in some of which there was still a certain amount of discharge. The sequestra, removed by operation, were put through rather an elaborate anaerobic technique. Although no definite examination for tetanus was carired out, it was probable that the presence of any anaerobes of the gas-forming or spore-bearing type might indicate, as was the case when colon bacilli were found in water, the presence of organisms of a more virulent type. Tetanus bacilli, therefore, were probably present in the majority of the cases of bone infection.

One of the points observed in the experiments was that anaerobic flora tended to increase in the bone sequestra while it decreased in the soft tissues. Another point was that a higher percentage of anaerobes was obtained in the old cases (those over 120 days) than in the newer ones. In the flora of the soft tissues, however, the case was just reversed. Apparently, then, anaerobes tended to disappear from the soft tissues, but to persist within the bone. The speaker described the technique by which he obtained the flora of the soft tissues and the flora of the bone.

He believed that tetanus persisted as well as the commoner organisms of the anaerobic group. Many cases of late tetanus were reported in which the onset followed secondary operations for bone grafting or reduction of old fractures. A case had been reported in which a man with an old fracture, going out for a walk for the first time, developed late tetanus. The speaker believed that such cases were probably due to the breaking down of callus or sequestra, with perhaps a slight hemorrhage, which furnished a new focus in which the newly liberated bacilli might start to grow and to secrete toxins.

Along this line, another experiment was performed. It was intended to show the relation of the condition of the wound to the onset of tetanus. Operative incisions were made in the thigh muscles of guinea-pigs and small bits of cloth satured with an emulsion made up with garden soil implanted. In one series of 6 animals a small bit of muscle tissue was separated and reimplanted with the cloth in the wound. In a series of 9 other guinea-pigs, great care was exercised not to injure the muscle more than was necessary to make the insertion of the cloth and to close the wound. Out of the 6 guinea-pigs in which detached muscle was reinserted, all died of tetanus. Out of the 9 guinea-pigs in which only the incision wound was made, but one died, and that death occurred at a fairly late period. It appeared, therefore, that the presence of devitalized tissue in a wound, is an important factor in causing the development of tetanus after inoculation with the tetanus bacillus.

The speaker agreed with Major Robertson, that practically every wound contained tetanus bacilli. The frequency and the severity of tetanus, therefore, was not mainly a question of presence of the bacillus. It depended rather on the resistance that might be created by antitoxin, or even more upon the nature and the condition of the wound.

Major Gibson, U. S. R., asked Sir William Leishman if the views he had expressed regarding the inefficacy of the intraspinal route were merely a difference of opinion among the authorities, or whether they were the result of convincing experiments. He had hoped that in the intraspinal method we had found an efficient means of administering serum. He wanted to cling to this belief as long as he could. He wondered if some of the failure, by this method had not resulted from too small doses, that is, from the hesitancy to push the treatment in the face of very severe reactions.

Major Lambert remarked that many of the anaphylactic rashes from tetanus antitoxin might be due to the peculiarities of the individual horses from which it was obtained. In producing diphtheria serum 25 years ago in New York, the authorities had observed that the serum from one of the horses never caused rashes. Antitoxin from other horses, however, caused such fearful rashes that they had to be discredited. The same was true for the tetanus horses which he had known.

Major Young, U. S. R., asked what the rules were regarding injections for trench feet with lesions, and also ulcerating skin diseases such as scabies.

Major Cushing, U. S. R., said that in April 1915 he had been shown a case of tetanus at the Hôtel Dieu in Paris as a great rarity. Tetanus, in fact, had practically disappeared as a result of serum prophylaxis. It was his impression also that in the British Army, likewise, there was little if any recognized tetanus at that time. At present, however, there was a good deal of the so-called delayed or local type of tetanus, and the speaker agreed with Major Blake in believing that all cases of persistent local spasm in a wounded extremity should rest under the suspicion of being examples of local tetanus. Due to the failure to recognize this condition as one of local tetanus, a death had occurred in the service of Base Hospital No. 5. A good many other examples of local tetanus had come under observation.

He and his colleagues were inclined to believe that the initial prophylactic dose of from 500 to 750 units had not been large enough. He thought that in the interest of our troops the question of whether a single dose of 1500 units was preferable to repeated doses of a smaller amount given at weekly intervals, as in the Bristish Army, ought to be definitely settled. It would be much easier in dealing with large numbers of wounded, to administer the single large original dose rather than to go to the labor of repeating the smaller. The speaker agreed with Major Gibson that general experience had been in favor of the intrathecal method when the disease was once established, and he wondered whether the size of the lumbar puncture needles which were employed might not possibly account for the unpopularity of this form of treatment. In some hospitals where he had worked, it was invariably the custom to give a general anesthetic before performing a lumbar puncture.

The speaker also asked Colonel Leishman whether a wound of the soft parts in which a large nerve was evidently divided was not the sort of wound in which it was advisable to give a large initial dose of antitetanic serum. He mentioned the case of a man who died on the tenth day following an abrupt attack of trismus. The projectile had divided the spinal accessory nerve and death occurred six hours after the onset of symptoms of the cranial type of tetanus. This man had received only 500 units as an initial dose.

One objection to the repetition of the doses was the possibility of upsetting a more or less seriously wounded and ill patient who was otherwise doing well. The speaker had seen two or three cases in which a second dose of serum had led to very disturbing symptoms, and one hesitated to add to the gravity of many patients' conditions by the chance upset which repetitions of serum might produce.

MAJOR MURPHY said that he feared he had conveyed a wrong impression by stating that he thought certain local spasms he had observed were not cases of local tetanus. He did not mean to imply that there was no such thing as local tetanus, but merely, to suggest that some cases which were supposed to be local tetanus were in reality only muscle spasms. He was inclined to believe that the omission of the second dose within the specified five days was the important point in Major Cushing's discussion. He requested Major Leishman to state how much the initial dose actually protects. He had supposed that in wounds of a certain type repeated doses were necessary in spite of a large initial dose.

SIR WILLIAM LEISHMAN, in replying to the questions raised, first took up the question of repetition of the prophylatic dose as to whether there was evidence that it was really necessary. There was not yet any definite statistical evidence that the second, third, and fourth injections had given any higher protection than that due to the initial dose. The evidence on this point was more likely to be obtained in England as comparatively few of the wounded were retained in France long enough to receive there the 3rd and 4th doses. Up to the present neither Sir David Bruce's figures nor the speaker's own were large enough to demonstrate any increased benefit from the repetition of the dose. At the same time there had been a certain number of cases of tetanus in France which had developed within the last four or five months in which the second and third doses had been given and several of these had been fatal.

As regards the influence of the incubation period upon the severity of the attack, the shorter the incubation period the more severe the attack. At the same time the influence of this factor had not, in the experience in France, been as great as one had expected to find it; there had been recoveries after an incubation of 4 or 5 days, and deaths in cases with very long incubation periods. The average incubation period in inoculated cases had been eight or nine days. On the other hand, in England there had been a very marked lowering of the mortality in cases which developed after a long incubation as compared with those which developed within 10 days of the injury. Again, it was probable that the generally lower rate of mortality at home hospitals, as contrasted with that in hospitals in France, was due to their including a larger proportion of cases of local tetanus and that many of these in their turn were of long incubation and were limited in their effects because of the influence of the prophylactic dose.

As regards intraneural injections the speaker said he had known of a few cases, but they had not been accompanied by favourable reports. Experience was, however, not large upon this point and, as a method, it was very little practised by British surgeons.

As to methods of treatment other than the specific use of antitoxin, the speaker said that the two chiefly tried earlier in the war were magnesium sulphate, by the various alternative methods, and carbolic acid in the various strengths that had been advocated, but both of these methods had bit by bit been dropped and unquestionably, in British experience, they had both been disappointing. Magnesium sulphate had certainly a controlling effect on the spasms, but it seemed to have no influence upon the progress of the case and was now very rarely used. Carbolic acid appeared to have been completely abandoned.

Practically all cases were treated by sedatives such as chloral, morphia and bromide, and there were some who attached greater importance to pushing these sedatives up to almost poisonous dosage than others. On the whole the speaker thought the general opinion was that these drugs were useful, but that they did harm if pushed too far.

With regard to the apparent change of opinion as to the value of the intrathecal route from that expressed in the first edition of the Official Memorandum on the Treatment of Wounds, published early in the war, the speaker said that such a change of opinion was a fact, but that it was a personal fact, as he must own to the authorship of the section in question.

As to whether there had been any anaphylactic shock, it was certain there had been cases, but they had been very rare, and none had resulted from the re-inoculation of men previously wounded. There had been some deaths during the course of an attack and they had usually followed on the injection of the serum intravenously, and in one case intrathecally.

As to the occurrence of tetanus in cases of ulceration of the skin, scabies, etc., this probably happened now and then, but it was difficult to be certain as to the facts and occasionally one got cases of tetanus in which the most careful search and enquiry revealed no evidence of previous injury or damage. On the whole it was surprising that such cases were not more common in view of the prevalence of tetanus spores in the soil.

Local tetanus was not as common in France as it appeared in the reports from home hospitals. The Tetanus Committee in

England had rightly called attention to this subject and to the importance of detecting as early as possible any signs of local spasm in or near the wound or of other local symptoms which might give early warning of tetanus and enable one to commence serum treatment as promptly as possible. But local tetanus which remained local had been rare out there and its comparative frequency at home was probably the result of the prophylactic dose which modified what would otherwise have been a general attack. The mortality of these local forms of tetanus appeared to be practically nil.

Major Cushing's sugestion about the advisability of employing a smaller needle for lumbar puncture was interesting, but the speaker did not think that it was the technique of the operation that had led some of the British surgeons to view this treatment with disfavor. It was rather because they had been disappointed with the results of the injections. Whatever the eventual judgment as to the value of intrathecal inoculations, it would at least be agreed that if they were to do good they should be given very early, and hence the importance of a close watch for the earliest signs of the disease.

Surgeon-General Thompson, D. M. S., First Army, at the beginning of the second meeting requested the opportunity to correct a wrong impression conveyed during the discussion of tetanus on the previous day. He had heard the statement made that the preventive dose of antitetanic serum was usually administered about 24 hours after a wound was received. Such a delay was exceptional. The dose was usually given within two or three hours. The orders were that it should be given at the advanced dressing stations, as soon as a man could be got there from the line. Of course, there were many exceptions, as when a man was wounded near to the German lines and might have to remain until nightfall before he was brought in. It was sometimes especially difficult to get the men out. In the recent fighting, it was said to have taken on an average about six hours to get the men to the dressing stations. Such instances, however, were not normal. The usual time was about two hours. There was a kind of tacit agreement on many parts of the First Army front, and the enemy did not shoot at men being carried on stretchers. In such cases the bearers were able to go over the top and avoid waste of time through trench carriage. As soon as a man received his injection, he was marked on the wrist with a larg " T ". In this manner, men who had failed to receive the dose were quickly detected at the C. C. S., and were inoculated there before they got any further.

Men who had been lying in the mud, or who had a very severe wound, were always given a double dose injected near to the wound, between the wound and the heart.

The walking wounded generally went to a separate collecting place. They were given the antitoxin even before their dressing was adjusted, and before they received their nourishment.

At the second meeting of the session, at 10 A. M., Jan. 15, Scabies and Pediculosis were discussed.

A paper written in French by Professor J. DARIER, Médecin-major, Hôpital St. Louis, Paris, was read in translation by the chairman. Parasites like the louse, it was stated, are exceedingly common in the French armies, and together with the acarus of scabies, they cause a majority of the pyodermias. It is always to be feared that the louse may become an agent in the spread of some serious epidemic like typhus and the plague.

In the army zone, pediculosis is said to be more frequent than scabies. The writer, however, has observed in his clinics (chiefly in Paris) that out of 100 patients with skin diseases, there is an average of about 15 cases of venereal diseases, 47 of scabies, and about 4 of pediculosis. Among 2000 men in the course of two years the author has observed 100 skin affections. Of these 40 o/o were scabitic. Pediculosis is exceedingly contagious, and may be communicated indirectly by bedding.

Pyodermia from lice is brought on in one or two ways : either by scratching occasioned by the itching, or by the actual infection from the parasite's bite, or from its crawling over the excoriations. (Test tubes containing peptone agar were exhibited showing an abundant growth of staphylococcus left behind by the lice which were allowed to crawl over the surface.)

Of the three common varieties of lice (*pediculus pubis*, *pediculus capitis*, *pediculus vestimenti*) the *pubis* variety, although more common than before the war, occasions but a small amount of pyodermia. The writer finds that the old mercury or gray ointment remedy often causes *dermatitis medicamentosa*, and thinks that it should be given up. He has employed instead a yellow oxide of mercury (yellow oxide 10, salicylic acid 1, vaseline 90). This preparation has sufficed to kill the lice and their nits, and has caused no inconvenience. The *pediculus capitis* is not commonly found among soldiers, and may be dealt with easily by clipping the hair.

The *pediculus vestimenti*, however, is very common. It occasions pyodermia of the ecthymatous type. The author states that he can confirm the fact announced simultaneously by Bulliard

(*Ann. de Derm.*, July 1917) and by Lemon and Barber (*l. c.*) that nits of the body louse are frequently to be found on the pubic hairs, as well as upon those of the axillae.

In deciding upon a method of treatment, the writer believes that not only the efficacy, but also the inoffensiveness, the rapidity, the practicability, and the cost, must be taken into consideration, especially because of the vast numbers of men to be dealt with.

In well equipped hospitals a satisfactory method of dealing with cases of pediculosis, is to sterilize the patient's clothing under pressure of steam while he takes a sulphur bath. He is directed to rub the hairy regions with an antiparasitic lotion. Preparations formerly used are costly or dangerous. Recently the *Assistance Publique* of Paris has recommended a preparation which gives satisfactory results. It is made up of trioxymethylene 1, benzine 10, vaseline 1000. Since, however, the bedding is not disinfected, the men are quickly infected upon their return to camp.

If sterilizers and baths are not available, the patient may be given a good soaping with hot water and chloroform water, and applications of salve in the hairy regions may suffice by way of sterilization and treatment. Shaving of the hair would he advantageous, but is repugnant to the men.

Of the many volatile preparations used, the writer has found the most satisfactory to be one made up of anisol 0.30, denatured alcohol, and distilled water, each 500. Volatile substances, however, have only a slow and incomplete effect upon nits, and must be used for 5 or 6 successive days. If clothes could be pressed with hot irons, the treatment would be considerably hastened.

Captain McCormac, R. A. M. C., read a paper in which he insisted upon the seriousness of scabies in reducing the man power of an army. He recalled that in the American Civil War out of 600,000 men there were reported 32,000 cases of itch besides 35,667 cases of unclassified skin diseases, and that in the Napoleonic campaigns, the cases of scabies were counted by the hundred thousand. The presumption of a high incidence in the present war had been fully realized.

Of the two parasites causing most of the dermatological affections, namely, the acarus and the louse, the louse, because of its visibility, had attracted most attention. The speaker thought this emphasis unfortunate, because of the practical impossibility of controlling the migrations of the louse. The itch parasite or acarus, however, clung tenaciously to the individual and his clothing, and others were not likely to become infested unless they wore the clothing or slept in the blankets of the person carrying the parasite. Prolonged contact with the individual or his clothing was necessary.

The limitation of scabies, therefore, appeared to be easier than the control of pediculosis. This was an especially fortunate circumstance because scabies was a much more harmful malady than pediculosis.

It must be admitted, however, that maximum efficiency was not yet reached in dealing with scabies. One source of weakness had been the fact that the batallion medical officer was generally appointed fresh from civilian practice in which he had little experience with scabies in the form observed in the army. He thus often overlooked it entirely.

The speaker gave in detail the characteristics of scabies as seen in the army. He stated that lesions on the hands were often wanting, and that when they were present vesicles were infinitely more common than burrows. The acarus could be extracted only with the greatest difficulty so that clinical rather than microscopical diagnosis had to be relied upon. When secondary impetigo occurred it might be of so severe a nature as to mask the underlying causal disease. This impetigo, even when widely spread, selected especially the lower buttocks, elbows, and knees, and upon this distribution a certain diagnosis could be made. Sometimes the pyodermic complication appeared in the form of boils. Hence in all cases of impetigo or boils, the medical officer should never fail to seek for confirmatory scabietic lesions on the penis, anterior axillary folds, wrists, etc.

Another difficulty met with was the fact that a soldier rarely reported until secondary impetigo had supervened. The itch he naturally attributed to lice until boils or ecthyma appeared. This delay not only prepared trouble for himself, but made him a disseminator of the disease among his mates.

It was clearly the duty of the medical officer to equip himself with a detailed knowledge of the disease, and then to inspect regularly not only the hands and wrists alone, but the whole body. All those found to be infected should be removed from the unit. He should insist especially that the blankets and clothing be properly disinfected. These were the chief source of the disease. Blankets were freely interchanged and underclothing might sometimes not be washed and sterilized as carefully as was desirable. He should be on the lookout also against the arrival of new cases.

*Treatment.* To avoid loss of time, centers for scabies treatment ought to be far forward. A station for each corps appeared most satisfactorily to fulfil all the essential requirements. It should be staffed by medical officers with special knowledge.

Of all the remedies successfully employed, sulphur, because of its cheapness and its efficaciousness, might be placed above all others. It could be employed as an ointment, a lotion, or in the form of a vapor. An ointment was easy to apply and sure in its results. The use of solutions required skill and was usually not thoroughly carried out. Sulphur vapor was open to the same objection made against it a century ago by Hebra. Although it often killed a proportion of the burrowed acari as well as those on the body, and therefore gave the appearance of a complete cure, it nevertheless might leave a few parasites still alive. A female, it was known, might live for from two to three months in her lair, laying several eggs a day. Men so treated might therefore become dangerous scabies carriers.

In applying the treatment the burrows and vesicles should first be laid open. A prolonged soaping with soft soap should be followed by a prolonged soaking in a warm bath. This bath is solely for the purpose of opening the burrows, and not for the purpose of cleansing or disinfecting. Inunction of the whole body below the neck with sulphur ointment on three successive days then serves to destroy the ova and acari without producing dermatitis. All infected clothing and bedding should be sterilized at the end of the treatment to prevent reinfection.

If secondary pyodermia once supervened, it required on an average 31.7 days for cure. It was therefore clearly imperative to seek out cases in the early stages when cure was comparatively simple.

The speaker expressed the hope that the American army might be spared a high incidence of this affection as a result of the increased knowledge of the disease and of the methods of handling it, acquired by British experience.

Captain Parkinson, R. A. M. C., who was next asked to speak, said that so much had recently been published on the subjects of scabies and pediculosis, and kindred skin diseases, that he could do no more than give the views of a great many other observers regarding the signs and diagnosis of these affections. He preferred to confine his attention to methods of preventing the spread of these diseases, with a view to drawing up rules for a campaign, especially against scabies.

He first called attention to some of the difficulties attending diagnosis. He had found the so-called " goose skin " very typical of scabies. He had noted, however, that a great many cases were atypical, not corresponding exactly to the types of the disease represented in pictures. Frequently scabies and pediculosis overlapped, so that it became almost impossible to distinguish them.

At the scabies dépôt, after a man was seen by the examining specialist, and the case pronounced scabies, he was given sulphur ointment treatment. The speaker said he was convinced that there was a sulphur dermatitis. He was not sure that it was due merely to overtreatment, although he had observed cases develop after a four days' sulphur treatment without rest.

The method of administering treatment at present was as follows:

A man was sent to the bath-house. His clothes, in the meantime, were sent to be disinfected. The disinfection was completed while the man was having his bath. The bath was of the utmost importance. Small baths were useless. The man should be submerged for twenty minutes. After the bath the sulphur ointment should be applied not by the man himself but by orderlies or other patients instructed for this purpose. If patients were employed they should be informed that there was no danger of contracting the disease themselves. At the end of twenty-four hours the man should be given another bath and the ointment once more applied. He was then given a rest for 24 hours. Then another treatment was given. The intervening periods of rest without treatment, as given in the First Army, had proved to be most essential. All treatment should be carried out in the presence of a medical officer and a case book showing treatment kept. Men that appeared cured, and who were sent back to service, often returned for treatment, and so a great wastage occurred. If they were kept long enough for a complete cure in the first place, such loss to the service could be avoided. With regard to baths, the scrubbing and opening up of the burrows were even more important than the germicide used.

The sulphur vapor treatment had been tried, but was now abandoned. Sulphur ointment, which was cheap and plentiful, was found to be the most effective method of dealing with scabies.

The speaker said that often the surroundings in the dépôts for the treatment of scabies, had not been made as cheerful as they were in the clearing stations, where every effort was made to make them as pleasant as possible. Scabies was essentially a war disease. The men suffering from it deserved as much consideration as those suffering from other diseases. Furthermore, the nature of their malady was such as to make them loose their self respect very easily and become discouraged. Everything possible was now being done to give men at the scabies dépôt the same comforts as sufferers from other diseases.

Taking up the subject of I. C. T. (inflammation of connective tissues) the speaker reviewed the experience of one English division. For eight months it remained in Egypt and was free from this complaint. It was then transferred to Gallipoli where an epidemic of I. C. T. broke out among the men. Later it returned to Egypt. Instead of living in billets as before, the division camped out in the desert, but still the I. C. T. continued. Apparently only the exposed parts suffered. The men wore knickers, and their legs were chiefly affected. Finally the division was removed to France, and here the infection persisted, although its incidence was considerably less. In some units where bathing and inspection were more frequently carried out than in the others, the I. C. T. was not nearly so prevalent. Undoubtedly the condition of cleanliness was of the greatest importance.

If it was true, as Captains Liman and Barber had remarked, that the eggs of body lice were found on the pubic hairs, it might be found necessary to shave these hairs in addition to the other methods of treatment. The speaker said he had searched carefully since reading an article announcing this observation, but he had so far been unable to find eggs on the hairs of the body.

The methods of treating scabies had been discussed in most armies. It was now generally thought best to have a separate center for the treatment of scabies. It was highly important to have a staff of specialists to deal with this disease, for unless the medical officers were interested in this special type of disease, there was little use in trying to treat it.

Not a very elaborate equipment was necessary at these stations. Well arranged baths and uniformity of treatment were the essentials. With a staff of specialists and of orderlies trained carefully as assistants, there was less likelihood of the maltreatment that sometimes occurred.

The speaker insisted upon the need for frequent inspection and thorough care of the men in a division. Inspection, of the old sort, as carried out in pre-war days, in which a hundred men were examined and required only to open their shirt fronts and show their wrists and fingers, was useless. It had been found that manifestations of scabies on the hands and wrists did not occur as frequently as might be supposed. A thorough examination, therefore, should be instituted as soon as the men come into rest billets, and it should be carried out daily. The body should be exposed to the waist, and at the same time, the condition of the teeth, general nutrition, and bodily cleanliness should be noted. When at rest, a company should be inspected each day.

To aid in the detection of the disease, it was highly important to have a trained man as orderly at the baths. He should be instructed in recognizing the signs of skin disease, and especially scabies. It was then possible for him to recognize early cases of scabies, which could be brought under treatment at once.

It was important to have good baths for the divisions. Most of the baths now established were good. They should be situated near the front and also farther back in the rest areas. Spray baths were not enough. While the man was bathing, his clothing should be rapidly but thoroughly disinfected. The hot air method, which Captain Jacobs was to describe in the afternoon, was found to be rapid and most effective. Clean underclothing should also be supplied after the bath, but great care must be exercised that the dirty clothes should not come in contact with the clean ones given out at the bath.

There could be no doubt that scabies was spread by blankets as well as by personal contact. The blankets, therefore, of a whole unit should be put through the disinfector while the unit was in the rest area. Otherwise, scabies would probably spread through out the unit. The authorities were endeavoring to have installed in all baths the apparatus to be exhibited by Captain Jacobs in the afternoon.

It was also necessary to gain the confidence of the men in the work of the laundry. They often preferred to do their own washing rather than put on clothing which possibly contained vermin or the eggs of vermin.

It had been found useful to boil the clothes in a solution of cresol, salt, and water in the proportion of 8 galls. cresol, 8 lbs. salt, and 800 galls. water. There had been an unwise tendency to neglect the washing of socks, especially now that great efforts were being made to prevent trench feet by giving out dry socks every day. Socks should be washed as carefully as the underclothing.

Attempts to disinfect by ironing had proved a failure. A squad of forty ironers would be required to iron the clothing of forty men all bathing at once. It was hardly ever possible to have more than one or two boys delegated for such work.

Another point often neglected was the disinfection of the lorry that conveyed the clothing to and from the laundry. It should be thoroughly disinfected before clean clothes were put into it. It would be still better to have a separate lorry for the purpose.

Although the health officers in England were very careful to prevent the scabies being brought in by men from the front, it had been observed that men returning to the front from England often brought fresh disease with them.

Care should be exercised in the scabies station to keep men in groups according to the length of time they had been under treatment. Otherwise a man just coming in might reinfect a man who had been treated for several days and who had nearly recovered.

It was very useful to keep charts showing skin diseases and to record statistics concerning the men coming from behind the lines, as, for instance, from schools, instruction camps, etc. It had been observed that scabies often moved from the back area to the front area. Every case of scabies admitted to a field ambulance should be accompanied by a report showing the dates of the last two skin inspections. In this way some idea might be obtained as to the manner in which these inspections were being carried out.

It was not always easy to tell when a man had recovered completely. As the acarus was difficult to find it should not be relied on as a test. Cases were sometimes discharged before the cure was complete. There might also be sulphur dermatitis causing irritation and suggesting that the disease was still active. In general, however, the treatment carried out along the lines mentioned above, had been effective, and comparatively few cases had to return for further treatment.

Dr. James C. Johnston said he believed there was great danger of overtreatment of scabies. This was true in civil as well as military practice. There was also a danger arising from the difficulty of diagnosis, mentioned by the previous speaker. Scabies is easily confused with chronic urticaria or prurigo. It was important, then, that the young medical officers should be carefully instructed with regard to the various types of skin reaction as well as parasitic disease. To this end, they should be brought in contact with as many cases as possible. They should be able to recognize not only scabies and pediculosis, but also the inflammatory diseases. Generally surgeons recruited from the medical profession in America had only a slight knowledge of dermatology. It would be an excellent thing for medical officers to have a two weeks' training, if it could be arranged, in the hospitals of Paris, particularly at Hôpital St. Louis, where daily cases came in literally by the hundreds. The speaker believed that Professor Darier and his colleagues at St. Louis would be willing to assist in training the American officers.

The speaker said he had little to add on the subject of treatment except to remark that at Hôpital St. Louis, one treatment with the ointment had been found sufficient instead of the three or four mentioned by the preceding speaker as essential. The men were given a rub with soap for fifteen minutes and then a hot bath for half an hour. During the bath they rubbed themselves carefully with brushes between the toes and fingers. They were then given the ointment, which they applied to each other, standing in a picturesque ring. This method of treatment certainly worked well. A physician or carefully trained orderly stood by to see that the work was done well. In the meantime their clothes were sterilized by steam, and were returned to them moist. The following day they were given a second bath and the treatment was complete. The speaker remarked that the French experience with ironing had been quite the reverse of the British experience as stated by Captain Parkinson. The French had found ironing by far the most desirable method of dealing with the outer garments.

On the subject of complications from overtreatment the speaker said that these were not as a rule so easy to handle as pyodermias and animal parasitic diseases. Cutaneous reactions were more difficult because generally the men prone to skin reactions like eczema were suffering from intestinal fermentation. He believed that if Balsam of Peru might be obtained for the armies, the treatment of scabies would lead to much better results. Since it was expensive its use might be confined to those cases in which there was dermatitis.

The speaker remarked that he was astonished at the great diversity of opinion as to methods of prophylaxis such as ironing the outer garments, sterilizing the underclothing at the laundries, etc. He believed that those methods which seemed reasonable, ought to be thoroughly tried out. Men should be looked after both going and coming, especially troups returning from a furlough. In Paris, scabies had become three times more common during the war than previously. A man might go from the trenches clean, and after a visit to Paris return with a case of scabies. The speaker thought that inspection at the front should be carried out once in eight days. The eggs of the itch mite often hatched in 8 days. If men were inspected every eight days instead of ten, as at present, relapses might be more quickly detected.

The speaker said he feared that the men in the trenches with all the prophylactic measures to which they were subjected, like the antitoxin injections, dusting powders, etc., were reaching the limit of endurance. He did not believe it was necessary to shave their hair, but he thought it might be clipped.

A good though idealistic suggestion had been made by Lieutenant Ferrand, surgeon at a French hospital base. He believed that baths should be installed close behind the lines so that there would be a separate bath for every thousand men. Each establishment might provide for from five to six bathers at a time. There should be a permanent staff of attendants. There should be a sterilization plant attached for disinfection by sulphur dioxide. Twelve men could be passed through in an hour. Only those with symptoms of itching from animal parasites need be put through the process. Naphthaline could be dusted over their bodies and inside their clothes at the same time.

Professor Darier said that he had found scabies generally to be a venereal disease. It was even more venereal than syphilis. Wives and husbands were likely to communicate it to each other, and children sleeping with their mothers often contracted it. It might, of course, be communicated by clothing. Men frequently contracted it when at home on a furlough. At the Hôpital St. Louis the question "Do you sleep alone?" was always asked. Generally the treatment had to be given for two or three persons from the same household.

The speaker said that he would be glad to welcome any American doctors who cared to visit his hospital where treatment for scabies was given in separate baths intended for the purpose. The treatment began daily at 9 : 30 A. M. and lasted for an hour and a half. Six or eight visitors desiring to observe the method might be received each day. First the men were given a thorough rubbing with soap. This was one of the most important factors in the treatment. It opened up the burrows and made the bath and the ointment applications more effective. After the rubbing the men were given a prolonged soaking in the bath. The sulphur ointment was then applied. During the period of treatment the men's underclothing was thoroughly sterilized by steam. It had not been found necessary to give the treatment more than once.

Surgeon-General Thompson, D. M. S., First Army, said that, in his army, every scabies patient was, on admission, given a hot bath; then he was well rubbed with sulphur ointment. On the second day, he had a second bath which washed off the remains of the sulphur. The skin was then allowed a twenty-four hours' rest. On the third day, a second cycle of treatment was given. Four such cycles were generally sufficient to cure an uncomplicated case. Some skins might stand more prolonged treatment, but there had been so many cases of dermatitis that orders were now given that twenty-four hours of complete rest from treatment should intervene between every two sulphur rubbings. When necessary zinc ointment might be applied in the intervals. The whole treatment required eight days : four days for the sulphur applications and four days of rest.

On the question of ironing, the speaker said that most of the louse eggs were at the fork of the trousers under the little piece of linen sewed over the rough seams. It was useless to iron unless this piece of linen was torn away. If this was done, the men complained of the discomfort afterwards. Furthermore, it took from fifteen to twenty minutes to do the ironing for each man. A kilt took three hours to iron thoroughly. The speaker had found it wholly impossible to do this thoroughy at the baths. He had therefore given out irons to the billets so that the men could do the ironing themselves. In any case it was practically impossible to get the ironing thoroughly done. With squads arriving every half hour for a bath hundreds of ironers would be required. Ironing had therefore been abandoned as being impossible to carry out on a large scale. Captain Jacobs had devised another method which he was going to demonstrate, and which seemed to promise some success.

At the third meeting of the Session at 2 P. M., January 15, the subject was the Louse Problem. Before this subject was taken up Captain Edward B. Krumbhaer, U. S. R., presented a paper read by Major Norris, U. S. R., on " A Pyogenic Gram-Negative Diplococcus Occurring in Skin Infections "[1].

The author stated that several times during the last six months a gram-negative diplococcus had been obtained at his hospital in apparently pure culture. So far as he had been able to ascertain, it was unlike any known pathogenic organism. Because of its possible occurrence in the spinal fluid in cases of traumatic meningitis, it should be carefully distinguished from the meningococcus. It might he differentiated by agglutination tests and differing sugar reactions.

The speaker stated that it had been isolated from infected scratches and gunshot wounds. The infection showed a tendency to burrow under the skin at the periphery of the infected area.

*Characteristics.* — It had been found mostly in pairs, and more often intra-cellular. It might be found isolated or in short chains. It was about the size of staphylococcus aureus, spherical, or slightly flattened on adjacent sides. It grew readily, but not luxuriantly, on plain agar slants, with a characteristic moist, milky white, opaque growth, tending to discrete round colonies. The

1. To be published elsewhere organism grew best aerobically at incubator temperature (37.5°C.). Under anaerobic conditions the growth was almost as great. It produced no gas or indol, but formed acid freely in both glucose and maltose broth. Litmus milk was slowly decolorized and sometimes partly coagulated. Gelatin was not liquified. The majority of the strains tested remained alive on agar for over two months. In fresh subcultures on agar it caused abcesses when injected subcutaneously, but failed to kill when injected intravenously or intraperitoneally. Similar colonies were occasionally found on plates from diarrheal stools, but it could not be shown that they were identical.

Captain Jacobs, R. A. M. C., read a paper on " Delousing by Dry Heat Treatment ". He stated that the prevalence of the louse had made it the most annoying insect the soldier had to contend with. Peacock had found in 1916 that 95 o/o of the men examined were infested with body lice. The female he found layed 5 eggs at a sitting and could lay a total of 125 eggs. They hatched in from 7 to 10 days and reached sexual maturity when they were 12 days old. The insect lived from 7 to 8 weeks under favorable circumstances and fed by sucking blood twice a day. Lice might survive apart from the body nearly 9 days. The home of the body louse was under the folds of the clothing, those next to the trunk being preferred. Most eggs were found at the fork of the trousers and this was because the trousers were worn for a much longer period than the shirt. The parts where most eggs were found, in the order of importance, were : the fork of the trousers, the arm pits, the junction of the tail of the shirt with the front flap, the trousers generally, shirt seams, and the neck.

The lice, however, were not confined to the part of the clothing nearest the host. Live lice and eggs had been found at the back of the neck of the tunic and the flap seams of the pockets and also in cardigan jackets. The insect was found in the largest numbers where there was warmth, humidity, and shelter. Dugouts, trenches, and billets were not important sources of dissemination. Blankets, empty sandbags, straw, and beds in the dugout, carried the pest, but when the articles were removed from the dug-outs, etc., these places ceased to be centers of distribution.

For the purpose of delousing it must be remembered that the soldier himself was the source of infestation. Lice spread by contact, crawling from kit to kit, or from soldier to soldier. Among the most important methods of killing and preventing lice from breeding, he mentioned : (1) chemicals, (2) steam, and (3) dry heat.

The dry heat method was the one now adopted in the British forces. Formerly, when a unit came out of the trenches it bathed at one of the Divisional bath-houses, and it was given a clean change of underclothing and the khaki clothing was hot ironed. The underclothing in the meantime was disinfected in the Corps or Divisional laundry by steaming at a pressure of 5 lbs. per square inch in a Foden Lorry " Thresh " for half an hour at a temperature of 220° F. This killed the lice and nits if the garments were not too tightly packed.

The khaki clothing was dealt with differently. While the men were bathing, their khaki clothing, which had previously been labeled, was handed into the ironing room and the seams were hot ironed. This was generally done by a number of men told off for this work and their instructions were to hot iron all the seams of the clothing and the forks of the trousers. Lice accumulated in large numbers under the piece of lining at the fork and this lining had to be removed before efficient ironing could take place. Hot ironing was a very slow process and as a bathing party should be dealt with in a quarter of an hour, it was necessary that the number of ironers should equal the number of bathers in a party. As there were usually 40 men in a bathing party, there was need for at least as many ironers. Not a quarter of this number were ever available. The result was that ironing was never efficiently done.

A means then had to be devised whereby clothing could be disinfected efficiently with a small outlay of labor. Experiments were conducted with dry heat. It was known that lice and nits were killed when exposed for 15 minutes to a temperature of 52° C. It had been demonstrated that exposure in water or dry heat at a temperature of 55° for half an hour was sufficient to destroy both active lice and nits — boiling for 5 minutes had a like effect. Experiments were therefore carried out in a hot air chamber especially designed for the tests. Lousy clothing was obtained and hung in the chamber, which was heated to a temperature of 60° C. The clothing was examined at varying intervals. After 10 minutes all lice were dead. After 20 minutes exposure, the clothing was removed and pieces of fabric containing clusters of nits were cut out and sent to a bacteriological laboratory for examination. It was reported that all the lice eggs were dead.

Tests were next made to ascertain whether articles of clothing were damaged by exposure to the heat, also articles commonly carried in the pockets such as tobacco and cigarettes. Specially severe tests were given to rubber articles. It was found that no appreciable deterioration took place.

A delousing chamber was then constructed according to the plan reproduced on page 237. It was given the following load of verminous khaki and clothing :

| | |
|---|---|
| Greatcoats | 47 |
| Tunics | 60 |
| Trousers | 57 |
| Drawers | 2 |
| Caps | 36 |
| Total | 202 |

The exposure to heat was for periods of 20 minutes and upwards. The initial heat was 82° C. and the lowest indicated, 70°. All lice and their eggs were killed.

*Suggested Scheme for Working* — In each Divisional area two delousing chambers with bath-houses should be built. There should be a permanent staff to obviate the necessity of new personnel with the consequent loss of continuity and efficiency. One of workable size could delouse the kits of 80 men per hour. It would include blankets, greatcoat, khaki, and underclothes. The time required for disinfecting the kit of 40 men was half an hour, 10 minutes being allowed for loading and unloading. Each batch of clothing would then be subjected to a temperature not lower than 60° C. for 20 minutes.

With two such establishments in each Division, disinfection might be carried out once a fortnight. About 1,440 men could be accommodated each day. All clothing, except the boots, should be surrendered upon entrance, and a check given, which during the bath, should be placed with the boots, in a numbered pigeon-hole provided for the purpose. Upon entering the dressing room, each man should be given clean underwear, and by this time the disinfected clothing would be ready.

*The structure of the delousing chamber* was described as follows : " The chamber has been designed so that it may be built entirely from material available under active service conditions. The structure is of 4 in. × 2 in. timber, lined with corrugated iron, sheet iron, or Uralite sheeting and rough-boarded on the outside, the space between being filled with sawdust. The floor is cemented to facilitate cleansing. The runners for the racks are angle iron pickets suspended from the ceiling by 2 in. × $^5/_8$ in. W. I. straps. The racks are 2 in. × 2 in. cross battens with 5 in. wire nails driven through the battens and bent up for hooks. Small 2 in. wire iron wheels, a local purchase costing 2 d., are fitted to make easy running. "

The speaker recommended that the chamber should be heated to at least 71° C. to allow for the drop of temperature due to the opening of the doors and the charging with unheated clothing. It was best to allow about 5 minutes between the entries of the racks. The doors should be left open as short a time as possible. The fire box ought not to be overfed with fuel if the best results were to be obtained. Dampers were supplied in the flues for the purpose of regulating the temperature. Wet articles should be first dried in a wholly different chamber, as a different system of ventilation is required. The furnace should be of cast iron with a heating surface of 1 square foot to each 100 cu. ft. of space to be heated, exclusive of the flue pipes. The flue pipes should start with one 6-foot length of 6 in. diameter cast iron piping. The remainder could be the ordinary 6 in. sheet iron piping. The main stack should be 9 in. in diameter. The radiant heat could be better diffused if a perforated sheet of iron were placed over the stove, leaving a space of 3 in. all around. Special attention should be paid to the ventilation of the chamber to prevent stratification of the heat. The heat drawn to the bottom of the chamber by having the fresh air delivered into the chamber from both sides of the fire box passing along underneath the combustion chamber to the center of the room and carried up 15 inches above the level of the floor. The ventilating ducts were so arranged in the cavity of the wall, that they drew off the air from each of the lower corners of the room. Thence they conducted the air to the main ventilation shaft, constructed from 2 in. × 1 in. battens braced, and the outlets covered with tarred felt. The whole was suitably stayed with iron wire to the superstructure. The outlet and inlet ducts should be of approximately the same size, i. e., one square foot for every 500 cu. ft. of space in the chamber. The shaft should be at least twice the height of the chamber. The consumption of coal was found to be from 7 to 10 lbs. per hour. A squad of 4 men in addition to the bath-house squad, was needed to manage the disinfecting chamber.

Captain Jacobs exhibited a device which served as a thermometer in the hot air chamber. It consisted of two sealed glass tubes filled with paraffin wax. The melting point in one tube was 62° C., the minimum temperature required in the chamber for practical working, and the melting point in the other tub was 75° C., or the maximum beyond which the clothing would be damaged. Black beads were placed on the paraffin in such a way as to fall when the wax melted. This device had the advantage over an ordinary thermometer, of being easily and quickly read at any time.

The speaker demonstrated the movement of the racks on wheels from the loading room through the doors into the heat chamber, and then through the doors on the opposite side into the discharging chamber. He said it was best to load only one rack at a time. In this way only one door need be opened at a time, and hence there would be less loss of heat. The speaker said that the chamber could, by the use of dampers, be converted into a drying room. He thought it better, however, to have separate establishments for this purpose. He said that the buildings were not strictly portable, but that they were so simple in construction that they were easily taken down and reconstructed.

LIEUTENANT MUELER, U. S. R., read a paper advocating the equipment of the men at the front with a pocket atomizer for spraying clothing. The model suggested is reproduced below. He believed that the most vigorous measures to prevent louse infestation were warranted, especially in the light of the growing belief that the louse is the natural carrier of trench fever. It had heen found in the British army that a powder made up of 66 o/o naphthaline, 2 o/o cresol, and 2 o/o iodoform was the most effective dusting preparation when applied to clothing. It had occurred to the author that since the naphthaline and cresol ingredients were obtained from distillate of coal tar, the crude liquid distillate might be employed as a spray. The cost would be less, and the liquid could be more evenly applied than the moist powder. He believed that a combination of creosote oil and heavy oil, both coal tar products, marketed in America under the name of creosote oil, would be most suitable. When sprayed, this preparation produced no harmful effects upon clothing, and even when there was a considerable concentration, only slight irritation of the skin resulted.

Atomizer for Delousing.

The atomizer, designed by Private Noble of General Hospital No 1, was simple and durable. It was operated by blowing. The reservoir contained about two ounces, sufficient for about a fortnight. It could be made of aluminum. The speaker had had only a very limited opportunity to try out the effect of the spray. He had done so with Sergeant Hamilton on a very small scale, and had found that by applications at intervals of several days, the worst case (in kilts) was completely freed of the vermin in 8 days. Apparently no eggs were alive at the end of this period.

Major Strong, U. S. R., said that in connection with the reports that had just been made he would like to ask several questions.

First, in relation to scabies : Had any of those who had spoken upon this disease any information regarding the prevalence and importance of albuminuria complicating it? Several observers had recently reported upon this complication. It had sometimes occurred in 10 o/o of the severe cases of scabies. Uhlenfield, in his thesis published in 1917, reports that in the study of 260 cases 6 o/o to 7 o/o had so called scaletic nephritis. It is supposed to differ in no respect from the ordinary exposure type of nephritis. The nephritis is supposed to be independent and not the result of treatment. It is presumed to be brought about by some irritating substance produced by the acarus scalei. The speaker asked if any of those who had spoken had followed its progress in their cases? It may be of interest in connection with trench nephritis.

In regard to the treatment of scabies, sulphur ointment had undoubtedly given the most favorable results in treatment in the war. The sulphur vapor bath had been tried extensively, but was not nearly so satisfactory or as practicable. One hears frequently that the cases are cured very speedily with sulphur ointment after proper soaping, scrubbing, and bathing, but when one visits the scabies hospitals one frequently sees cases that have been under treatment for much longer periods of time without cure, and one of the speakers has referred to the fact that it has become recognized now as more advisable to keep the cases under observation and treatment for a longer period of time than formerly.

Major Strong said he was glad to hear that Balsam of Peru had been mentioned by Dr. Johnston and he wondered if it would not be possible to obtain and import this substance for use in the armies and give it as thorough a trial as had been given sulphur ointment. It had often proved very effective in civil practice.

The statement which was recently published by Semon and Barber had been referred to by one of the speakers, namely, that *pediculus vestimenti* almost *invariably* attaches its eggs to the pubic hair. The speaker thought the statement was very important and he wondered if it would be accepted without reserve by all investigators. It was, of course, a very important point in connection with delousing, but it did not necessarily demand that individuals infected must be shaved.

In referring to delousing Major Strong said the disinfecting chamber just referred to and demonstrated by Captain Jacobs undoubtedly constituted an improvement on many other delousing plants. It was well known that temperature at 52 degrees C. for half an hour destroyed both lice and their ova; that a temperature of 65 degrees C. for one minute and of 62 degrees C. for five minutes also destroyed them. Water at 60 degrees C. for five minutes killed them. A number of different types of delousing plants had been devised which were very effective; whether we used hot air or steam for desinfection was not essential. It was a simple proceding to delouse large numbers of people. But it did no good to delouse say, half a division of troops and then put them with the other half that were still infected with lice; that pediculosis was very largely spread only by the infected individual; that successful delousing of troops was largely a question of proper organization and administration. Until there was proper organization and administration troops would continue to remain lousy.

With reference to the spray apparatus recommended by Lieutenant Mueller, he thought this might prevent infection in individual cases. However, the substance was volatile and after a short time the amount sprayed on the clothing would evaporate. It was true that the ova of the body louse hatched, under normal conditions, in from seven to twelve days, but under certain abnormal conditions the hatching might be delayed for much longer periods, even to five weeks, and the substance being volatile would no longer have any effect on the development of the young pediculi.

Captain Taylor said there seemed to be practically no difference of opinion as to the ease of delousing. It was comparatively a simple matter to kill lice and their nits. Only the administrative aspect of the problem presented any real difficulties. There were, however, many very simple means of killing lice and nits that ought not to create a serious administrative problem. For instance, it was known that a solution of 1 to 1000 cresol added to 1 to 1000 formalin would kill all lice and nits on socks and underclothing which were soaked in it for two hours. There was a very general agreement that the cresols were among the most effective and practical of delousing agents.

So far most of the delousing methods employed were directed to the killing of the louse, and not so much to the rendering of his habitat uncomfortable. The habitat was almost exclusively the inner clothing next to the skin, where the body heat and food made it necessary for the louse to live. If the underclothing were properly treated by such solutions as the one mentioned, the whole problem might possibly be dealt with in the rinsing water at the laundries.

In reply to Lieutenant Mueller's question regarding the supply of cresol in France, the speaker said that it was plentiful and easily obtained. It could be purchased for 18 centimes the kilo, or 1.25 francs the litre.

The speaker asked if any thorough test had been made among the British troops of the procedure recommended by Bacot in 1916. This was that the underclothing should be embrocated in a 5 o/o solution of an emulsion composed of 45 o/o to 50 o/o of soft soap combined by heating with 50 o/o to 55 o/o of crude carbolic. The speaker also recalled that an experiment reported favorably by Gunn in May 1917 had been tried on undergarments specially designed for the purpose. These were prophylactically treated by immersion in a solution of an ounce and a half each of naphthaline and sulphur added to one gallon of benzol or petrol.

Major Blake said that Captain Taylor had omitted to mention some experiments carried out with regard to the antisepsis of underclothing, using almost the identical materials recommended for treating clothing to prevent pediculosis. Captain Taylor found that clothing thus treated did not produce severe infections when carried into the tissues, and that therefore the treatment of underclothing by these methods might have not only a destructive effect upon vermin, but also a direct influence upon the infection of wounds. The most severe infections were not caused by the missile itself, but by bits of clothing carried into the wound. This was a matter of interest to the surgeon as well as the bacteriologist and should be seriously looked into. He would like to know if the treatment of underclothing had ever been taken up in a methodical way by the British or French armies.

Major Strong said Bacot had experimented particularly with crude carbolic acid in the treatment of clothing as a prophylactic measure against lice. He found that by impregnating the shirt with a solution of less than 2 1/2 o/o of crude carbolic or cresol the destruction of lice was doubtful. The strength which proved favorable was between 5 o/o and 10 o/o. The success depended largely upon the amount the individual perspired. If more than 5 o/o was used in summer the perspiration with the carbolic caused irritation of the skin. Treated flannel could be left 15 days before losing its efficiency.

Professor Darier referred to Major Strong's statement that albuminuria was observed in about 5 o/o of scabies cases, and said that this was the case only when there was secondary infection. He thought its occurrence was absolutely of no importance and was not a counter-indication for the treatment by sulphur ointment.

The speaker said that he had tried almost all of the methods advanced, and that he had found sulphur ointment, if methodically and thoroughly applied, to be by far the quickest, the cheapest, and the most successful treatment. It should always be preceded by a thorough bath with scrubbing.

He agreed that the hot-air method of disinfestation as described by Captain Jacobs was ideal. It required, however, an apparatus which was too elaborate to be easily available everywhere. Such plants could not always be at the disposal of the sanitary authorities. The same objection might be made to disinfection by gaseous substances.

Naphthaline seemed to be the most effective of the dusting powders used against lice. The addition of iodoform was quite useless.

Soaking of the clothing seemed to the speaker wholly impracticable. Spraying, however, was possible, but cresol used for the purpose gave the clothing a very disagreable odor. If the spray were to be used some other substance must be found. Anisol was pleasanter and it had the further advantage of being rather cheap. It was nearly as active as cresol.

The speaker said that he could confirm by personal experience the statement that the eggs of the louse were to be found on the body hairs. Since most men objected to having these hairs shaved, it was best to disinfect by ointment.

Major Lambert emphasized the fact that the purpose of the Research Society was not to report upon details which might be of individual or general scientific interest. The present society, unlike peace organizations, had the very definite object of saving man power for the army. It was its business to deal with facts as they were to be found in the actual life of the soldier. First it was important to know these facts as they has been observed by others, and upon the basis of this knowledge and experience to work out the best means of protecting our men who are over here in the struggle.

He said that it might be of interest to know that as a result of the previous meeting a Commission had been appointed for the study of trench fever, and that already the laboratory was completed and ready for the investigation. He said that he had received General Bradley's permission for the immediate action of the Commission.

Similarly a Committee under the regular army had been appointed to investigate gas gangrene. Dr. Bull, who had produced in New-York a powerful serum against the Welch bacillus, had arrived from England. People who had seen the effect of the serum in England and America were enthusiastic about it. Whether it would prove effective against gas gangrene remained to be seen. There could be no doubt, however, that a thorough investigation of its possibilities was a step in the right direction. In a similar way, it would be the purpose of every meeting of the society to bring about some practical endeavor for the conservation of our armed forces.

He announced the following make-up of the committees :

*Trench Fever* : Major Strong, Major Cushing, Major Swift; and, as Laboratory Staff, Major McNee, Major Swift, Captain Opie and Captain Baetjer.

*Gas Gangrene* : Colonel Wallace, Captain Henry, Captain Taylor and Major Murphy.

---

## Figures and Tables

**Figure f1:**
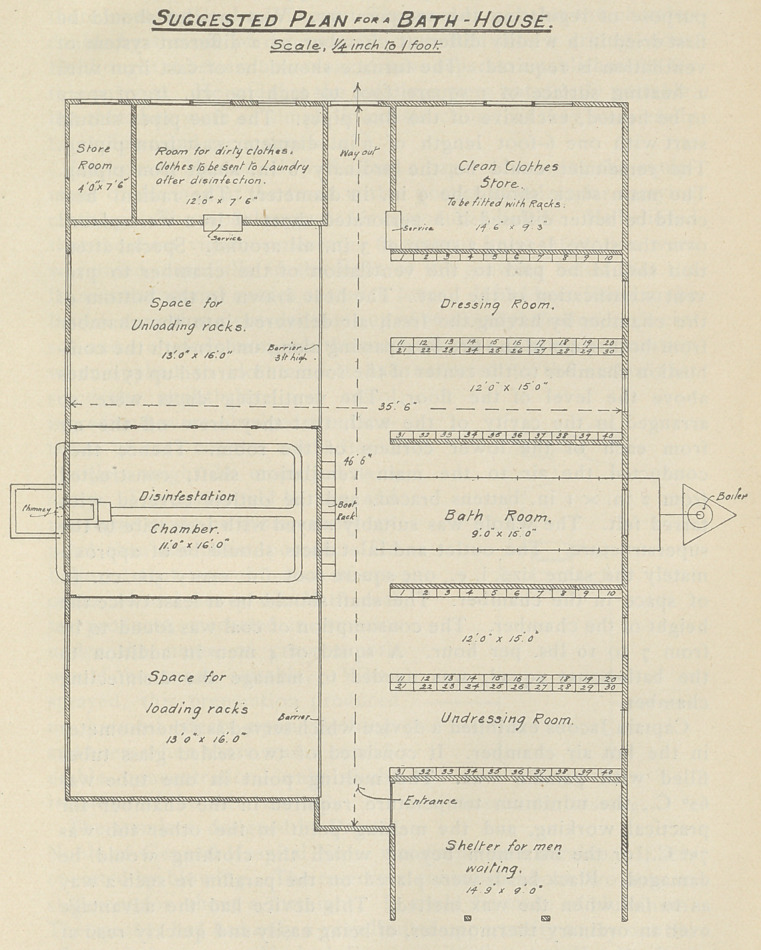


**Figure f2:**